# 303. Evaluation of Antimicrobial Utilization and the Incidence of Bacterial Pneumonia Co-infection in Non-ICU COVID-19 Patients at an Urban Academic Medical Center

**DOI:** 10.1093/ofid/ofab466.505

**Published:** 2021-12-04

**Authors:** Sara Groome, Claudine El-Beyrouty, Meghan Mitchell

**Affiliations:** Thomas Jefferson University Hospital, Warminster, Pennsylvania

## Abstract

**Background:**

The management of COVID-19 poses diagnostic challenges with regard to concomitant bacterial pneumonia. This may result in unnecessary antibiotic therapy. This analysis described the experience of an urban academic medical center’s management of non-ICU patients diagnosed with COVID-19 during the initial months of the pandemic and assessed the rate of concomitant bacterial pneumonia in this population.

**Methods:**

This retrospective analysis evaluated patients 18 years and older admitted to Thomas Jefferson University Hospital (TJUH) between March 1, 2020 and July 31, 2020 who had a positive COVID-19 test, were symptomatic, and received at least one dose of antibiotics. Antibiotic therapy was considered appropriate if there was objective evidence of bacterial pneumonia. Per the TJUH COVID-19 guidelines, objective diagnostic criteria assessed included the following: MRSA nasopharyngeal swab, urine *Legionella pneumophilia* or *Streptococcus pneumoniae* antigen test, respiratory pathogen panel, and sputum culture. If patients did not have evidence of bacterial pneumonia, the threshold for appropriate discontinuation of antibiotics was 48 hours.

**Results:**

50 patients were included in the final analysis. Upon admission, 7 (14%) patients had clear chest radiographs, and 9 (25%) of the 36 patients with a procalcitonin drawn had a level ≥ 0.25, indicating a potential bacterial infection. 15 (30%) patients were known to be COVID-19 positive prior to being administered antibiotics. Additionally, 22 (44%) patients had an infectious diseases service consult during their admission. 25 (50%) patients were continued on antibiotics > 48 hours. The mean duration of antibiotic therapy in the entire population was 3.4 days (82 hours). The monthly average duration of antibiotic therapy trended downward as the pandemic progressed. The most common empiric antibiotic regimen was ceftriaxone and azithromycin, received by 28 (56%) patients. Only 2 (4%) patients were diagnosed with bacterial pneumonia.

**Conclusion:**

In a sample of 50 COVID patients the overall rate of concomitant bacterial pneumonia was 4%. Given this finding, it is vital to remain judicious with the use of antibiotics and to employ the assistance of antimicrobial stewardship colleagues when managing patients diagnosed with COVID-19.

**Disclosures:**

**Claudine El-Beyrouty, PharmD, BCPS**, **Astellas** (Advisor or Review Panel member)**Shionogi** (Advisor or Review Panel member)

